# Supplementary figures and images for: Multi-Omic Analyses of the m5C Regulator ALYREF Reveal Its Essential Roles in Hepatocellular Carcinoma
Source: Front Oncol. 2021 Jul 23;11:633415. doi: 10.3389/fonc.2021.633415 (PMC8343179; doi:10.3389/fonc.2021.633415)

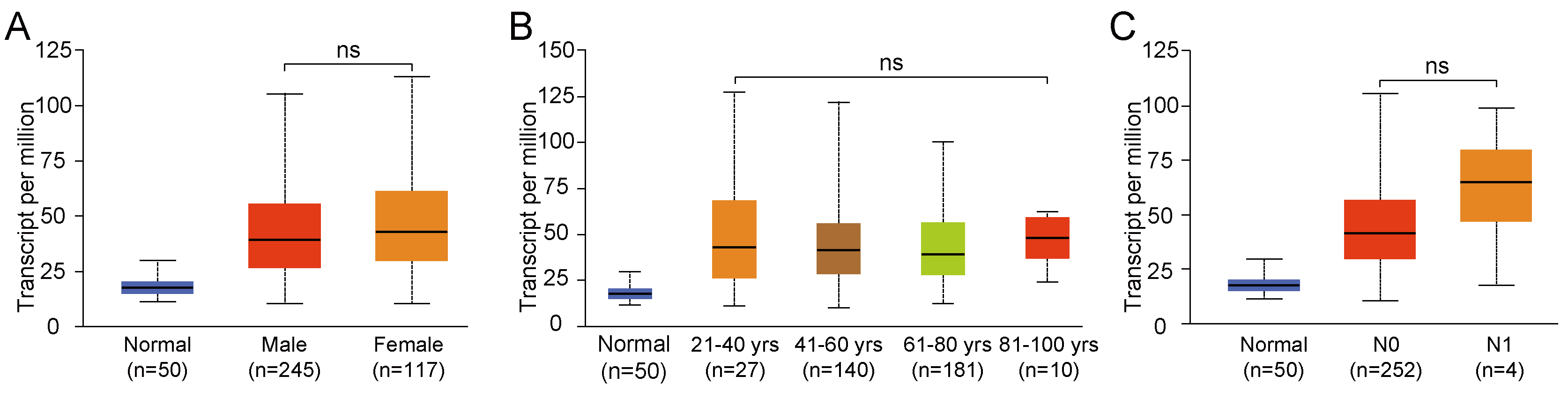

Supplement: Supplementary Figure 1 — ALYREF mRNA expression based on clinical-characteristic subtypes. (A–C) ALYREF mRNA expression showed no significant differences based on gender, age, staging, and early metastasis status. [file Image_1.tif]

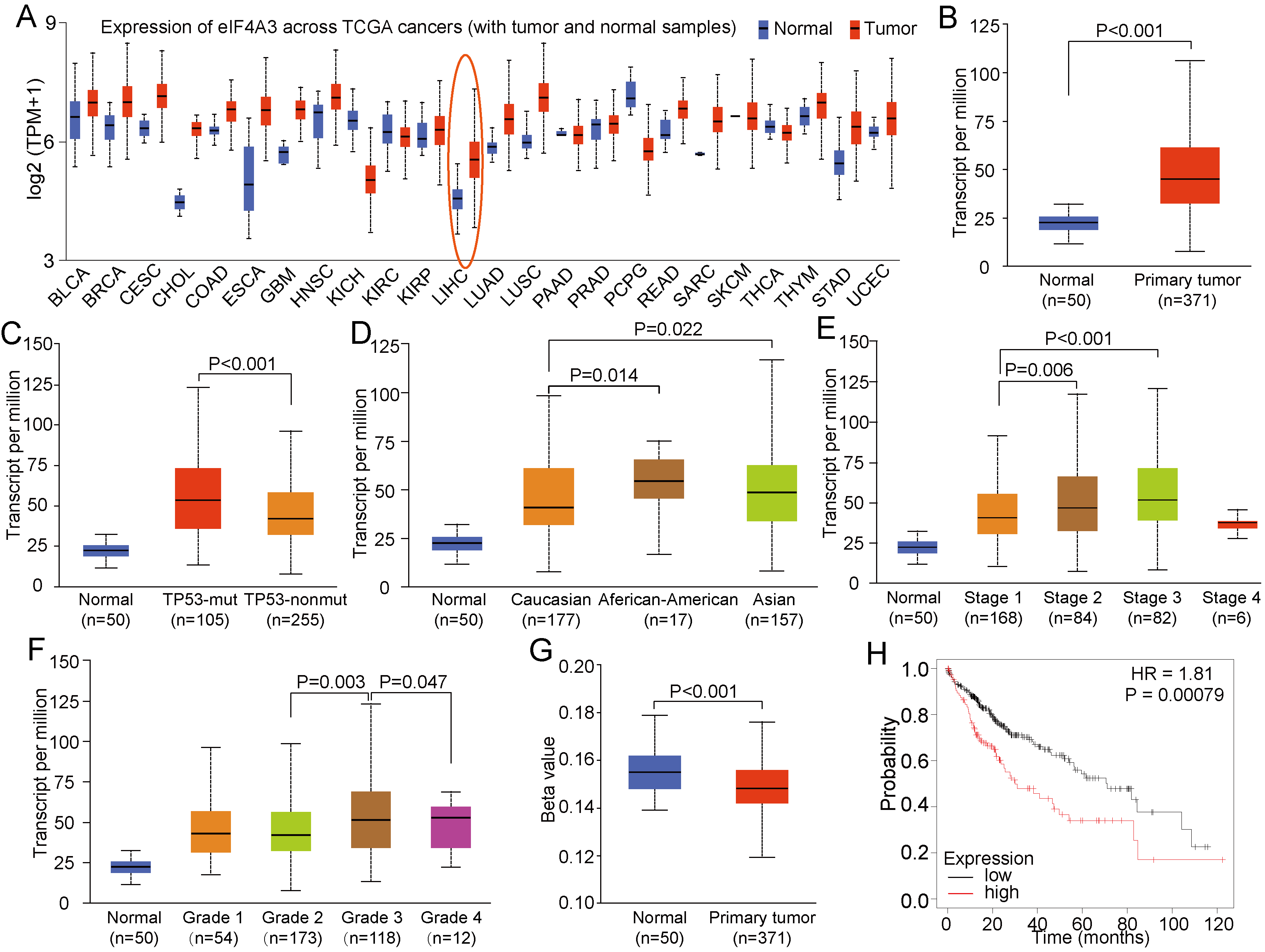

Supplement: Supplementary Figure 2 — Increased pan-cancer expression of eIF4A3 mRNA and in clinical characteristic-based liver hepatocellular carcinoma (LIHC) subgroups. (A) eIF4A3 mRNA expression was increased in pan-cancer tissues compared to normal tissues, including LIHC. (B) eIF4A3 mRNA expression was significantly increased in primary tumor samples compared to normal tissue samples. (C–F) ALYREF transcription levels in different clinical trait-based groups. The specific groupings were based on TP53 mutation status, race, clinical staging, and clinical grading. (G) The beta values for eIF4A3 mRNA expression in primary liver cancer and in normal samples. (H) A Kaplan-Meier analysis showed that high eIF4A3 expression indicated poor prognosis compared to low eIF4A3 expression. [file Image_2.tif]

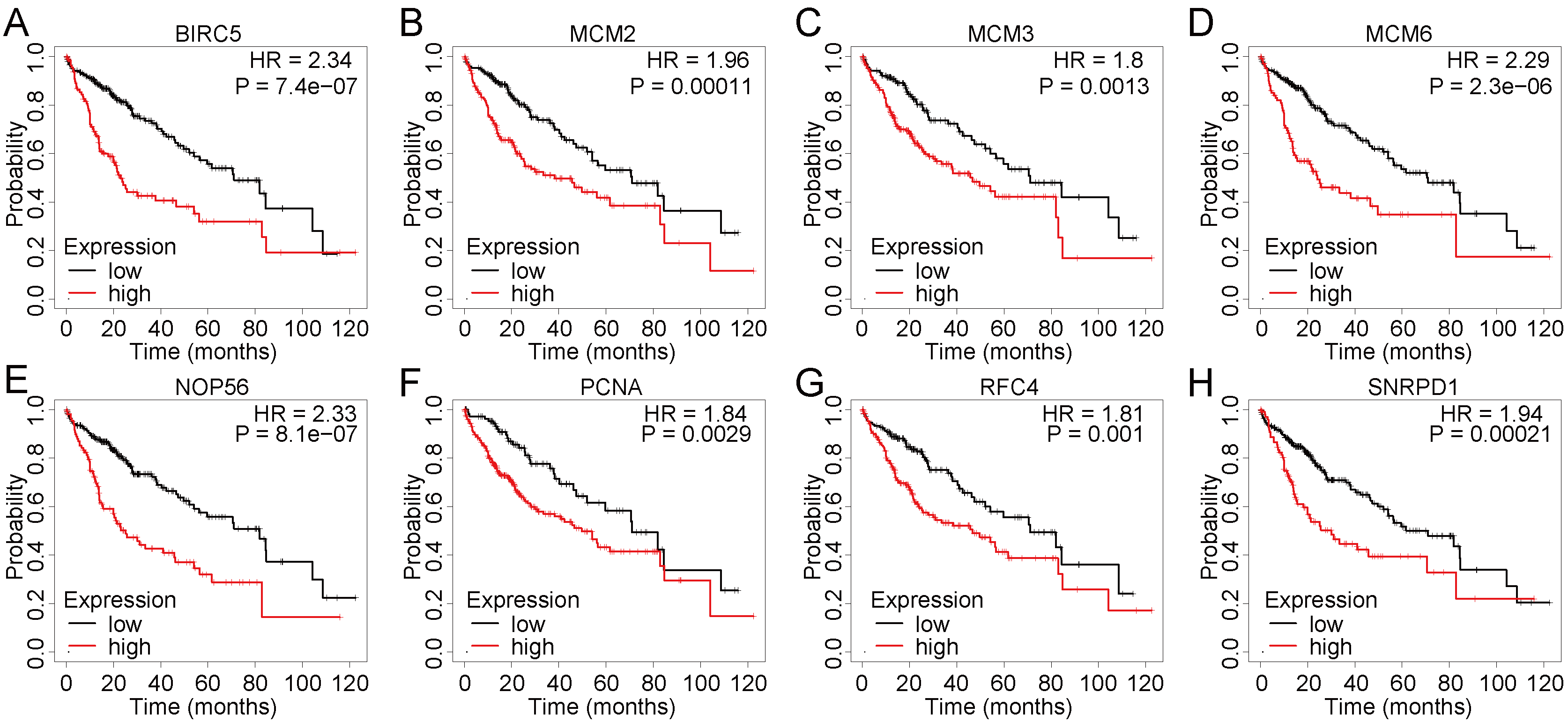

Supplement: Supplementary Figure 3 — A prognosis analysis based on the eight hub genes. (A–H) An overall survival analysis using the eight hub genes (BIRC5, MCM2, MCM3, MCM6, NOP56, PCNA, RFC4, and SNRPD1) showed that their high expressions were associated with poor prognosis compared to their low-expression subgroups. [file Image_3.tif]
